# An immunohistochemical study of TIMP-3 expression in oesophageal squamous cell carcinoma

**DOI:** 10.1038/sj.bjc.6602185

**Published:** 2004-10-05

**Authors:** T Miyazaki, H Kato, M Nakajima, A Faried, J Takita, M Sohda, Y Fukai, S Yamaguchi, N Masuda, R Manda, M Fukuchi, H Ojima, K Tsukada, H Kuwano

**Affiliations:** 1 Department of General Surgical Science (Surgery I), Gunma University Graduate School of Medicine, 3-39-22, Showa-machi, Maebashi, Gunma 371-8511, Japan

**Keywords:** oesophageal cancer, tissue inhibitor of matrix metalloproteinase 3 (TIMP-3), invasion, prognosis, immunohistochemistry

## Abstract

Tissue inhibitor of metalloproteinase-3 (TIMP-3) inhibits the activity of matrix metalloproteinase, which may play an important role in carcinoma invasion and metastasis. We have investigated the relationship between TIMP-3 reduction and clinicopathological factors in oesophageal squamous cell carcinoma (ESCC). We examined tissue specimens that had been removed from 90 patients with thoracic oesophageal cancer who had undergone surgery between 1983 and 2001. Immunohistochemical staining was performed by the standard streptavidin–biotin method. Immunostaining of TIMP-3 was seen in the cytoplasm of cancer cells and normal oesophageal epithelial cells, particularly in cells located in shallow areas of the tumour. TIMP-3 preserved (+), moderate (±), and reduced (−) cases accounted for 30, 27, and 33 of the 90 patients, respectively (33, 30, 37%). Significant correlations were observed between TIMP-3 expression and depth of tumour invasion (*P*=0.001), number of lymph node metastases (*P*=0.003), infiltrative growth pattern (*P*=0.003), and disease stage (*P*=0.005). The survival rates of patients with TIMP-3 (−) cancer were significantly lower than those of patients with TIMP-3 (+) and TIMP-3 (±) cancer (*P*=0.0003). The mean 5-year survival rates of patients with TIMP-3 (+), (±), and (−) were 50, 58, and 21%, respectively. In conclusion, decreased expression of TIMP-3 protein correlates with invasive activity and metastasis. This makes the prognosis for patients with cancer that has lost TIMP-3 significantly less favourable than that for patients with cancer that has maintained TIMP-3.

The extracellular matrix is an important structure in maintenance of tissue organisation and in the suppression of cellular proliferation and migration. Excess destruction of the extracellular matrix is associated with many pathologies, including atherosclerosis, rheumatoid arthritis, and cancer progression (Liotta *et al*, 1980; Tryggvason *et al*, 1987; Palletier *et al*, 1990). The intricate balance between net extracellular matrix deposition and degradation is controlled by a complex system of tightly regulated protease enzymes and their endogenous inhibitors. Matrix destruction is thought to be a key event in both the local invasion and distant metastasis associated with tumour progression, and genes that inhibit these processes may be useful for cancer therapy. Matrix metalloproteinases (MMPs), a family of Zn^2+^ metalloproteinases involved in the degradation of extracellular matrix macromolecules, are associated with tissue destruction under various pathological conditions. Previous experimental and clinicopathological studies have revealed a good correlation between expression of MMPs and invasive phenotype of tumour cells or frequency of metastasis (Stetler-Stevenson *et al*, 1993; Sato *et al*, 1994). Expression of MMP-1, -2, -3, -7, and -9 in oesophageal cancer has already been reported (Shima *et al*, 1992; Ohashi *et al*, 2000). In addition, Ohashi *et al* (2000) have reported the expression of tissue inhibitor of MMP (TIMP)-1 and -2, MT1-MMP, and MT2-MMP by immunohistochemical staining.

The TIMPs are a family of molecules that inhibit the proteolytic activity of MMPs, which also play an important role in tumour invasion and metastasis (Sato *et al*, 1992; Miyagi *et al*, 1995; Wang *et al*, 1997).

TIMP-3 is a secreted 24-kDa protein that, unlike other TIMP family members, binds to the extracellular matrix. Its functions have been reported as inducing apoptosis of cancer cells (Ahonen *et al*, 1998; Baker *et al*, 1999), and suppressing tumour growth and angiogenesis (Anand-Apte *et al*, 1996, 1997; Bian *et al*, 1996). Furthermore, TIMP-3 expression decreases at the invasive edge of poorly differentiated adenocarcinoma of the human colon, which suggests that a regional loss of TIMP-3 may contribute to their increased invasiveness (Powe *et al*, 1997). However, the relationship between TIMP-3 expression and clinical features (tumour invasiveness, metastasis and prognosis) in patients with ESCC is not known.

In this study, an immunohistochemical analysis of TIMP-3 protein expression was performed to determine the relationship between TIMP-3 reduction and clinicopathological factors in ESCC.

## MATERIALS AND METHODS

### Patients and tissue samples

The tissue specimens used had been removed from 90 patients with thoracic ESCC who had undergone surgery at Gunma University Hospital between 1983 and 2001. Written informed consent to participate in the study was obtained from each patient before surgery, according to the ethical guidelines of our university. All patients underwent potentially curative surgery without preoperative therapy. There were 76 men and 14 women, aged 40–78 years (mean age: 60.9 years). Tumour stages were classified according to the fifth edition of the TNM classification of the International Union against Cancer (UICC). Evaluation of tumour differentiation was based on histological criteria of the guidelines of the Japanese Society for Esophageal Diseases (1999). The mean postoperative follow-up period was 33.9 months (range: 6.2–192.2 months).

Specimens were fixed in 10% formaldehyde solution and embedded in paraffin. We examined sections that contained both a tumour-invasive portion and normal oesophageal epithelium.

### Antibodies

The monoclonal antibody (Mab) specific for TIMP-3 (clone 136-13H4) was purchased from Daiichi Fine Chemical Co. Ltd., Toyama, Japan.

### Immunohistochemistry

Immunohistochemical staining was performed by the standard streptavidin–biotin (SAB) method. Briefly, each 4-*μ*m tissue section was deparaffinised, then rehydrated and incubated with fresh 0.3% H_2_O_2_ in methanol for 30 min at room temperature. After rehydration through a graded ethanol series, the sections were autoclaved in 10 mM citrate buffer (pH 6.0) at 120°C for 5 min, then cooled to 30°C. After incubation with normal rabbit serum for 30 min, the tissue sections were removed by blotting. The sections were then incubated at 4°C overnight with anti-TIMP-3 Mab at a dilution of 1 : 800 in phosphate-buffered saline (PBS) containing 1% bovine serum albumin, then washed in PBS and incubated with secondary antibody for 30 min at room temperature. Immunohistochemistry was performed using a Histofine SAB-PO(M) kit (Nichirei, Tokyo, Japan). The chromogen was 3,3′-diaminobenzidine tetrahydrochloride, applied as a 0.02% solution containing 0.0055% H_2_O_2_ in 50 mM ammonium acetate – citric acid buffer (pH 6.0). The sections were lightly counterstained with haematoxylin. Negative controls were prepared by substituting normal mouse serum for primary antibody, and no detectable staining was evident.

### Evaluation of immunostaining for TIMP-3 expression

We evaluated immunostaining of cancer cells in all layers of the oesophageal epithelium. When more than 80% of carcinoma cells in a given specimen were positively stained as well as normal epithelium in the same section, the sample was classified as TIMP-3 preserved (TIMP-3 (+)); when less than 30% were stained, as TIMP-3 reduced (TIMP-3 (−)); and when 30–80% of cells were stained, as TIMP-3 moderate (TIMP-3 (±)).

### Statistical analysis

Statistical analysis was performed using unpaired two-group *t* test for age and number of lymph node metastases. The *χ*^2^ test was used to analyse the effects of sex, differentiation, location, and TNM clinical classification. Survival curves were calculated by the Kaplan–Meier method, and analysis was carried out by the log-rank test.

## RESULTS

### Relationship between TIMP-3 expression and clinicopathological features

**Figure 1:**
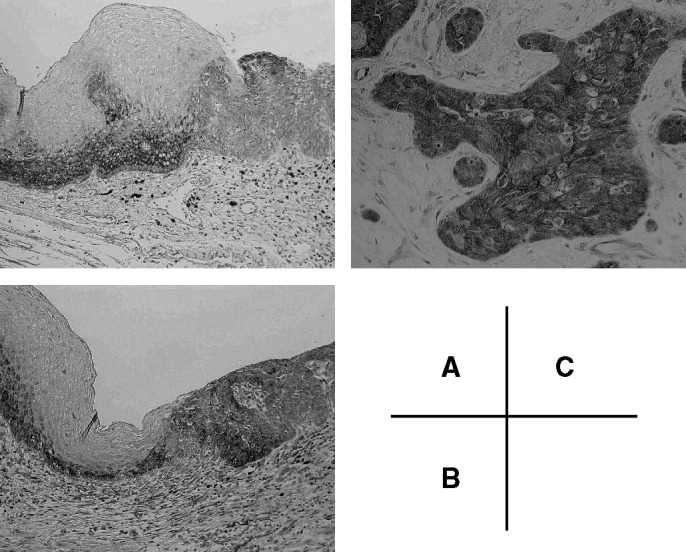
Representative photomicrographs of tissue sections immunostained for TIMP-3. (**A**) TIMP-3 was detected in the cytoplasm of the basal cells, parabasal cells, and leucocytes in normal oesophageal epithelium (left side). In this case, TIMP-3 expression in the cancer cells (right side) was weaker than that in the normal epithelium. This case was regarded as TIMP-3-reduced (× 100). (**B**) The arrowhead indicates primary oesophageal cancer with TIMP-3 protein expression (× 100). This case was regarded as TIMP-3 preserved. (**C**) High-power view of the immunohistochemistry. TIMP-3 was detected in the cytoplasm of cancer cells (× 200).

**Figure 2:**
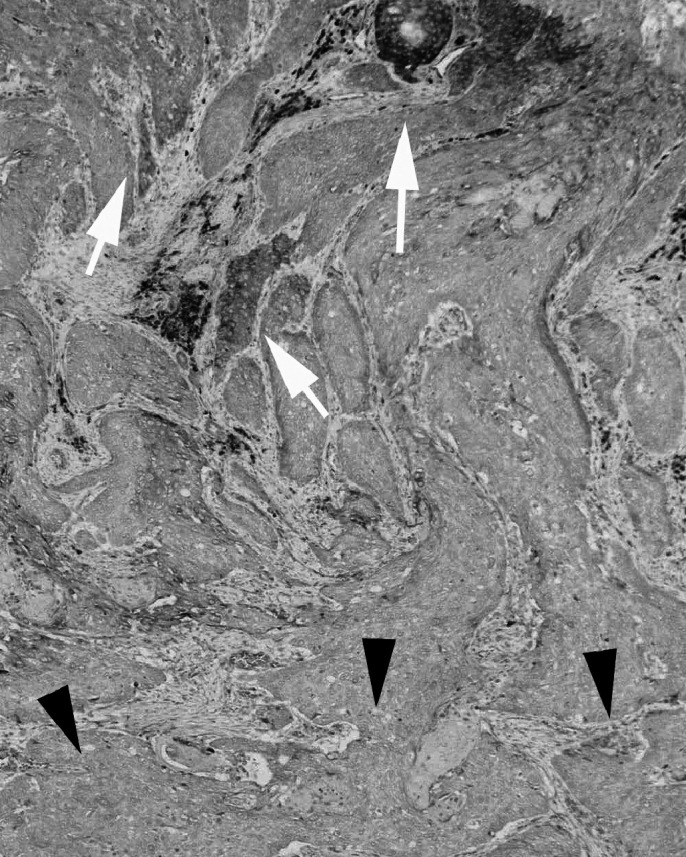
Representative photomicrographs of tissue sections immunostained for TIMP-3 (× 50). Black arrowheads indicate deep areas of tumour invasion. Cancer cells from these areas did not express TIMP-3 protein. White arrowheads indicate shallow areas of tumour, where cancer cells expressed TIMP-3 protein.

Tissue inhibitor of metalloproteinase-3 expression in ESCC was investigated by immunohistochemical analysis of formalin-fixed, paraffin-embedded specimens using a TIMP-3 specific Mab. In normal oesophageal tissue, immunostaining of TIMP-3 was detected in the cytoplasm of the basal cells, parabasal cells and stromal cells (Figure 1A, B

). Immunostaining of TIMP-3 was seen in the cytoplasm of all cancer cells (Figure 1B, C). Expression of TIMP-3 was preserved in shallow areas of the tumour, but it was reduced in deep areas of the same tumour (Figure 2

). TIMP-3 preserved, moderate, and reduced cases accounted for 30, 27, and 33 of the 90 patients, respectively (33, 30, 37%). The relationship between the clinicopathological characteristics of patients with ESCC and TIMP-3 expression is summarised in Table 1

**Table 1:** The correlationship between clinicopathological characteristics and TIMP-3 expression

		**TOMP-3 preserve**	**TOMP-3 moderate**	**TOMP-3 reduce**	
**Parameters**	**Total**	**(*n*=30)**	**(*n*=27)**	**(*n*=33)**	***P*–value**
Age (mean±s.d., years)	60.9±8.3	60.9±7.5	59.9±8.2	61.8±9.1	NS
					
*Gender*					
Male	76	23	23	30	
Female	14	7	4	3	0.295
					
*Differentiation*					
Well	22	7	6	9	
Moderate	45	15	15	15	
Poor	23	8	6	9	0.957
					
*Location*					
Upper	12	1	6	5	
Mid-thora	55	21	18	16	
Lower	23	8	3	12	0.060
					
*TNM clinical classification*					
T					
T1	34	20	9	5	
T2	13	2	6	5	
T3	37	8	11	18	
T4	6	0	1	5	0.001
					
N					
N0	36	16	11	9	
N1	54	14	16	24	0.108
					
M					
M0	74	28	21	25	
M1	16	2	6	8	0.147
					
*Stage*					
I	23	15	4	4	
II	28	7	12	9	
III	23	6	5	12	
IV	16	2	6	8	0.005
					
No. of lymph node metastases (mean±s.d.)	—	1.3±1.8	3.1±5.9	2.5±3.4	0.003
					
*Lymphatic invasion*					
ly (−)	28	13	7	8	
ly (+)	62	17	20	25	0.206
					
*Blood vessel invasion*					
v (–)	49	18	14	17	0.756
v (+)	41	12	13	16	
					
*Infiltrative growth pattern*					
inf *α*	22	14	2	6	
inf *β*	60	12	23	25	
inf *γ*	8	4	2	2	0.003
					
*Intraepithelial spread*					
ie (−)	45	14	10	21	
ie (+)	45	16	17	12	0.111
					
*Intramural metastasis*					
IM0	80	27	25	28	
IM1	10	3	2	5	0.619

s.d.=standard deviation.

. A significant correlation was observed between TIMP-3 expression and depth of tumour invasion (*P*=0.001), number of lymph node metastases (*P*=0.003), infiltrative growth pattern (*P*=0.003), and disease stage (*P*=0.005). However, there was no significant association with age, sex, tumour location, presence of regional lymph node metastasis, lymphatic invasion, blood vessel invasion, intraepithelial spread, intramural metastasis, or presence of distant metastasis.

**Figure 3:**
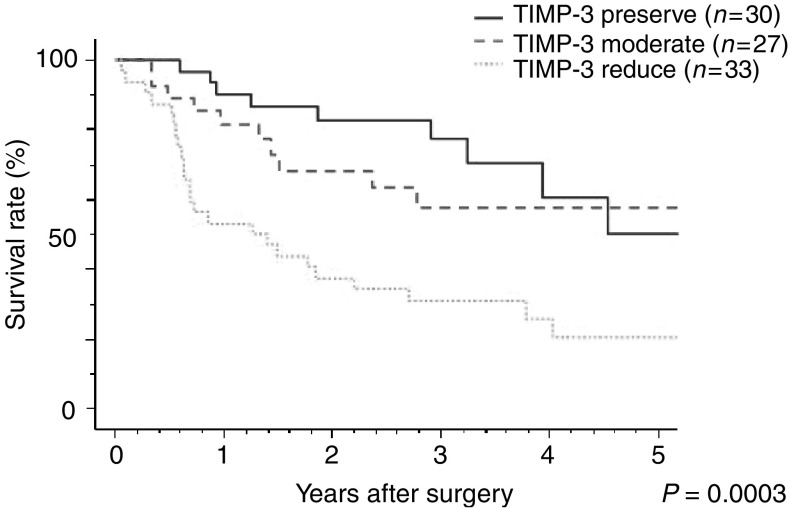
Relationship between overall postoperative survival and TIMP-3 expression. Patients with TIMP-3-reduced cancer had a significantly poorer prognosis than those with TIMP-3 preserved or moderate expression (5-year survival rates: TIMP-3 reduced, 21%; TIMP-3 moderate, 58%; TIMP-3 preserved, 50%; *P*=0.0003).

The survival rates of patients with TIMP-3 (−) cancer were significantly lower than those of patients with TIMP-3 (+) and TIMP-3 (±) cancer (*P*=0.0003; Figure 3

). The mean 5-year survival rates of patients with TIMP-3 (+), (±), and (−) were 50, 58, and 21%, respectively. Multivariate analysis showed that TIMP-3 overexpression was not a prognostic factor by itself, in contrast to depth of tumour invasion, lymph node metastasis, or disease stage (data not shown).

## DISCUSSION

Our immunohistochemical results suggest that expression of TIMP-3 protein is correlated with depth of tumour invasion, the number of lymph node metastases and disease stage. The expression of TIMP-3 protein was localised in shallow areas of the tumour, and was reduced in deep areas of the same tumour. In particular, no expression was observed at the invasive tumour front. This result indicated that invading cancer cells had lost their expression of TIMP-3. Furthermore, the prognosis for patients with cancer that had lost TIMP-3 was significantly less favourable than that for patients with TIMP-3 (+) and TIMP-3 (±) cancer.

Bachman *et al* (1999) showed that loss of TIMP-3 expression is associated with dense methylation of the 5′-CpG island in cell lines from many common human cancers. This methylation-associated silencing of TIMP-3 is tumour-specific, and associated with lack of TIMP-3 protein expression in primary cancers. Aberrant methylation of *TIMP-3* was demonstrated in primary cancers of the kidney, brain, colon, breast, and lung, but not in any of 41 normal tissue samples. Reduced TIMP-3 expression in ESCC may also have been caused by aberrant methylation in our study.

Baker *et al* (Ahonen *et al*, 1998; Baker *et al*, 1999) reported that TIMP-3 expression inhibits invasion and induces apoptosis in cancer cells. They used adenovirus-mediated gene delivery of TIMP-1, -2, and -3 to melanoma, cervical carcinoma, and fibrosarcoma cell lines. Their reports support ours. Tissue inhibitor of metalloproteinase-3 expression may be unfavourable for the survival of cancer cells, and cancer cells with a high grade of malignancy (more invasive and fewer apoptotic cells) may not express TIMP-3 for various reasons.

An imbalance between cell surface-associated proteinases and their inhibitors (higher concentrations of MMPs and lower concentrations of TIMPs) has been traditionally implicated in tumour expansion. However, it has been reported that some TIMPs function as growth factors (Hayakawa *et al*, 1992, 1994). For example, TIMP-1 and TIMP-2 have erythroid potentiating activity, and accelerate the growth of most cells (Hayakawa *et al*, 1992, 1994). Tissue inhibitor of metalloproteinase-3 has been reported to stimulate the proliferation of growth-retarded, nontransformed cells maintained under low-serum conditions (Yang and Hawkes, 1992). These reports do not coincide with our data, but growth activity is not always consistent with invasive activity.

Other functions of TIMP-3 have been reported, including suppression of tumour growth and angiogenesis (Anand-Apte *et al*, 1996, 1997; Bian *et al*, 1996; Spurbeck *et al*, 2002; Qi *et al*, 2003). Qi *et al* have demonstrated the ability of TIMP-3 to inhibit vascular endothelial growth factor (VEGF)-mediated angiogenesis and identified the possible mechanism involved. In ESCC, VEGF has been reported to be one of the most important angiogenesis factors (Shih *et al*, 2000; Kato *et al*, 2002). Therefore, TIMP-3 may suppress tumour angiogenesis indirectly by inhibiting the VEGF angiogenesis signal, and in turn, tumour growth.

Mori *et al* (2000)investigated TIMP-1 expression of mRNA and protein in ESCC, and reported that TIMP-1 expression is correlated with high-grade malignant behavior, and is an independent prognostic factor. The structure of the protein-coding exons is similar in the *Timp-1* and *Timp-3* genes (Apte *et al*, 1995). Moreover, TIMP-3 and TIMP-1 inhibition were quantitatively similar, implying that all TIMPs are equally efficient in MMP inhibition (Apte *et al*, 1995).

Decreasing expression of TIMP-3 in invasive oesophageal cancer cells may indicate that one of the functions of TIMP-3 is suppression of invasiveness. If cancers having invasive and metastatic ability do not express TIMP-3, preserving or increasing expression of TIMP-3 may lead to a novel cancer therapy. Matrix metalloproteinase inhibitor has recently been developed, and is undergoing trials as an investigational agent (Nozaki *et al*, 2003). Combination therapy with MMP inhibitor and TIMP-3 enhancement may be more effective than MMP inhibitor alone.

In conclusion, decreased expression of TIMP-3 protein correlates with invasive activity and metastasis. This makes the prognosis for patients with cancer that has lost TIMP-3 significantly less favourable than that for patients with cancer that has maintained TIMP-3.

## References

[bib1] Ahonen MA, Baker AH, Kaharl VM (1998) Adenovirus-mediated gene delivery of tissue inhibitor of metalloproteinases-3 inhibits invasion and induces apoptosis in melanoma cells. Cancer Res 58: 2310–2315 9622064

[bib2] Anand-Apte B, Bao L, Smith R, Iwata K, Olsen BR, Zetter B, Apte SS (1996) A review of tissue inhibitor of metalloproteinases-3 (TIMP-3) and experimental analysis of its effect on primary tumor growth. Biochem Cell Biol 74: 853–862 9164653 10.1139/o96-090

[bib3] Anand-Apte B, Pepper MS, Voest E, Montesano R, Olsen B, Murphy G, Apte SS, Zetter B (1997) Inhibition of angiogenesis by tissue inhibitor of metalloproteinase-3. Invest Ophthalmol Vis Sci 38: 817–823 9112976

[bib4] Apte SS, Olsen BR, Murphy G (1995) The gene structure of tissue inhibitor of metalloproteinases (TIMP)-3 and its inhibitory activities define the distinct TIMP gene family. J Biol Chem 270: 14313–14318 7782289 10.1074/jbc.270.24.14313

[bib5] Bachman KE, Herman JG, Corn PG, Merlo A, Costello JF, Cavenee WK, Baylin SB, Graff JR (1999) Methylation-associated silencing of the tissue inhibitor of metalloproteinase-3 gene suggests a suppressor role in kidney, brain, and other human cancers. Cancer Res 59: 798–802 10029065

[bib6] Baker AH, George SJ, Zaltsman AB, Murphy G, Newby AC (1999) Inhibition of invasion and induction of apoptotic cell death of cancer cell lines by overexpression of TIMP-3. Br J Cancer 79: 1347–1355 10188875 10.1038/sj.bjc.6690217 PMC2362728

[bib8] Bian J, Wang Y, Smith MR, Kim H, Jacobs C, Jackman J, Kung HF, Colburn NH, Sun Y (1996) Suppression of *in vivo* tumor growth and induction of suspension cell death by tissue inhibitor of metalloproteinases (TIMP)-3. Carcinogenesis (London) 17: 1805–1811 8824499 10.1093/carcin/17.9.1805

[bib9] Hayakawa T, Yamashita K, Ohuchi E, Shinagawa A (1994) Cell growth-promoting activity of tissue inhibitor of metalloproteinases-2 (TIMP-2). J Cell Sci 107: 2373–2379 7844157 10.1242/jcs.107.9.2373

[bib10] Hayakawa T, Yamashita K, Tanzawa K, Uchijima E, Iwata K (1992) Growth-promoting activity of tissue inhibitor of metalloproteinases-1 (TIMP-1) for a wide range of cells. A possible new growth factor in serum. FEBS Lett 298: 29–32 1544418 10.1016/0014-5793(92)80015-9

[bib11] Kato H, Yoshikawa M, Miyazaki T, Nakajima M, Fukai Y, Masuda N, Fukuchi M, Manda R, Tsukada K, Kuwano H (2002) Expression of vascular endothelial growth factor (VEGF) and its receptors (Flt-1 and Flk-1) in esophageal squamous cell carcinoma. Anticancer Res 22(6C): 3977–3984 12553021

[bib12] Liotta LA, Tryggvason K, Garbisa S, Hart I, Foltz CM, Shafie S (1980) Metastatic potential correlates with enzymatic degradation of basement membrane collagen. Nature 284: 67–68 6243750 10.1038/284067a0

[bib13] Miyagi E, Yasumitu H, Hirahara F, Minaguchi H, Koshikawa N, Miyazaki K, Umeda M (1995) Characterization of matrix-degrading proteinases and their inhibitors secreted by human gynecological carcinoma cells. Jpn J Cancer Res 86: 568–576 7622422 10.1111/j.1349-7006.1995.tb02436.x PMC5920870

[bib14] Mori M, Mimori K, Sadanaga N, Inoue H, Tanaka Y, Mafune K, Ueo H, Barnard GF (2000) Prognostic impact of tissue inhibitor of matrix metalloproteinase-1 in esophageal carcinoma. Int J Cancer 88: 575–578 11058873 10.1002/1097-0215(20001115)88:4<575::aid-ijc9>3.0.co;2-c

[bib15] Nozaki S, Sissons S, Chien DS, Sledge Jr GW (2003) Activity of biphenyl matrix metalloproteinase inhibitor BAY 12-9566 in a human breast cancer orthotopic model. Clin Exp Metast 20: 407–412 10.1023/a:1025473709656 14524529

[bib16] Ohashi K, Nemoto T, Nakamura K, Nemori R (2000) Increased expression of matrix metalloproteinase 7 and 9 and membrane type 1-matrix metalloproteinase in esophageal squamous cell carcinomas. Cancer 88: 2201–2209 10820340

[bib17] Palletier JP, Mineau F, Faure MP, Martel-Pelletier J (1990) Inbalance between the mechanisms of activation and inhibition of metalloproteases in the early lesions of experimental osteoarthritis. Arthritis Rheum 33: 1466–1476 2171538 10.1002/art.1780331003

[bib18] Powe DG, Brough JL, Carter GI, Bailey EM, Stetler-Stevenson WG, Turner DR, Hewitt RE (1997) TIMP-3 mRNA expression is regionally increased in moderately and poorly differentiated colorectal adenocarcinoma. Br J Cancer 75: 1678–1683 9184186 10.1038/bjc.1997.285 PMC2223522

[bib19] Qi JH, Ebrahem Q, Moore N, Murphy G, Claesson-Welsh L, Bond M, Baker A, Anand-Apte B (2003) A novel function for tissue inhibitor of metalloproteinases-3 (TIMP3): inhibition of angiogenesis by blockage of VEGF binding to VEGF receptor-2. Nat Med 9(4): 407–415 12652295 10.1038/nm846

[bib20] Sato H, Kida Y, Mai M, Endo Y, Sasaki T, Tanaka J, Seiki M (1992) Expression of genes encoding type IV collagen-degrating metalloproteinases and tissue inhibitors of metalloproteinases in various human tumor cells. Oncogene 7: 77–83 1311064

[bib21] Sato H, Takino T, Okada Y, Cao J, Shinagawa A, Yamamoto E, Seiki M (1994) A matrix metalloproteinase expressed on the surface of invasive tumor cells. Nature 370: 61–65 8015608 10.1038/370061a0

[bib22] Shih CH, Ozawa S, Ando N, Ueda M, Kitajima M (2000) Vascular endothelial growth factor expression predicts outcome and lymph node metastasis in squamous cell carcinoma of the esophagus. Clin Cancer Res 6(3): 1161–1168 10741747

[bib23] Shima I, Sasaguri Y, Kusukawa J, Yamana H, Fujita H, Kakegawa T, Morimatsu M (1992) Production of matrix metalloproteinase-3 related to malignant behavior of esophageal carcinoma. Cancer 70: 2747–2753 1451050 10.1002/1097-0142(19921215)70:12<2747::aid-cncr2820701204>3.0.co;2-5

[bib24] Spurbeck WW, Ng CY, Strom TS, Vanin EF, Davidoff AM (2002) Enforced expression of tissue inhibitor of matrix metalloproteinase-3 affects functional capillary morphogenesis and inhibits tumor growth in a murine tumor model. Blood 100(9): 3361–3368 12384438 10.1182/blood.V100.9.3361

[bib25] Stetler-Stevenson WG, Aznavoorian S, Liotta LA (1993) Tumor cell interactions with the extracellular matrix during invasion and metastasis. Annu Rev Cell Biol 9: 541–573 8280471 10.1146/annurev.cb.09.110193.002545

[bib26] Tryggvason K, Hoyhtya M, Salo T (1987) Proteolytic degradation of extracellular matrix in tumor invasion. Biochim Biophys Acta 907: 191–217 2823896 10.1016/0304-419x(87)90006-0

[bib27] Wang M, Liu YE, Greene J, Sheng S, Fuchs A, Rosen EM, Shi YE (1997) Inhibition of tumor growth and metastasis of human breast cancer cells transfected with tissue inhibitor of metalloproteinase 4. Oncogene 14: 2767–2774 9190892 10.1038/sj.onc.1201245

[bib28] Yang TT, Hawkes SP (1992) Role of the 21-kDa protein TIMP-3 in oncogenic transformation of cultured chicken embryo fibroblasts. Proc Natl Acad Sci USA 89: 10676–10680 1438264 10.1073/pnas.89.22.10676 PMC50404

