# Supplementary figures and images for: An immunohistochemical study of TIMP-3 expression in oesophageal squamous cell carcinoma
Source: Br J Cancer. 2004 Oct 5;91(8):1556–60. doi: 10.1038/sj.bjc.6602185 (PMC2409930; doi:10.1038/sj.bjc.6602185)

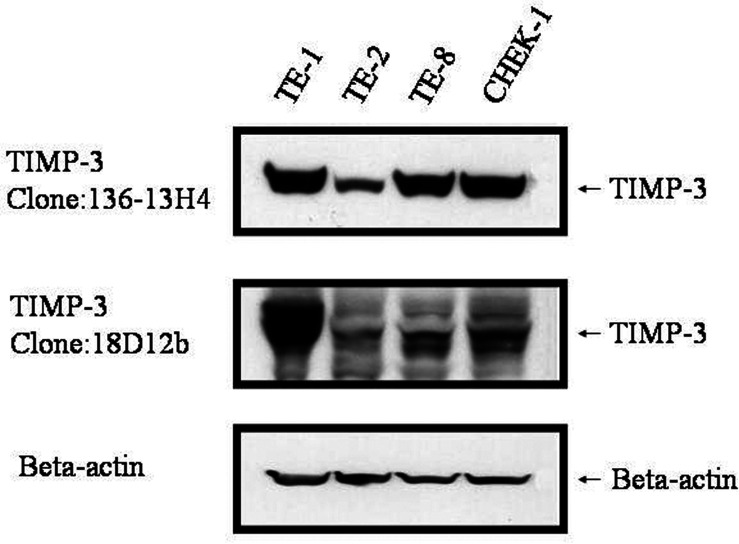

Supplement: Supplementary Information [file 91-6602185x1.jpg]
